# A Pilot of a Sustainability-Extending Intervention in Canadian Nursing Homes

**DOI:** 10.1093/geroni/igab046.2071

**Published:** 2021-12-17

**Authors:** Lauren MacEachern, Yuting Song, Liane Ginsburg, Adrian Wagg, Matthias Hoben, Malcolm Doupe, Carole Estabrooks, Whitney Berta

**Affiliations:** 1 University of Toronto, Toronto, Ontario, Canada; 2 University of Alberta, Edmonton, Alberta, Canada; 3 York University, York University, Alberta, Canada; 4 University of Alberta at Edmonton, Edmonton, Alberta, Canada; 5 University of Manitoba, Winnipeg, Manitoba, Canada; 6 University of Toronto, University of Toronto, Ontario, Canada

## Abstract

Understanding of intervention sustainability processes is limited. Failure to sustain evidence-based innovations means that intended improvements are short-lived, scale-up and spread are unlikely, and real losses are incurred on research investments. We explored the sustainability of a health care aide (HCA)-led quality improvement (QI) initiative, Safer Care for Older Persons (in residential) Environments (SCOPE), that was implemented in long-term care homes (LTCHs) in Manitoba, Canada. Based on our understanding of factors influencing post-implementation sustainability processes, we developed and piloted a “low-dose” and “high-dose” “Booster” intervention to extend the two-year post-implementation period over which SCOPE was naturally sustained. Both versions of the “Booster” involved the following components: a HCA-led team with management support, a workshop to review SCOPE QI approaches and tools, a binder of QI resources, and supports from an experienced Quality Advisor (QA). We collected data from various sources to depict the most accurate account of QI sustainability and conducted thematic analysis to understand each team’s experience with sustainability processes. We used a qualitative assessment rubric to evaluate the impact of the “Booster” conditions on the teams’ performance against core SCOPE components. Our results suggest that the “Booster” served to establish more relaxed expectations and generally renew interest in LTCH QI initiatives. The calibre of management support was associated with teams’ performance and management support varied with the level of QA support. These pilot results will inform the next study phase, which examines longer-term sustainability of QI initiatives in LTCHs beyond the initial 2-year post-implementation period.

